# Allow natural death versus do-not-resuscitate: titles, information contents, outcomes, and the considerations related to do-not-resuscitate decision

**DOI:** 10.1186/s12904-018-0367-4

**Published:** 2018-10-10

**Authors:** Sheng-Yu Fan, Ying-Wei Wang, I-Mei Lin

**Affiliations:** 1 Institute of Gerontology, College of Medicine, National Cheng Kung University, Tainan, Taiwan; 2 Department of Family Medicine, Buddhist Tzu Chi General Hospital, Hualien, Taiwan; 3 Department of Psychology, College of Humanities and Social Sciences, Kaohsiung Medical University, Kaohsiung, Taiwan

**Keywords:** Allow natural death, Barriers, Cardiopulmonary resuscitation, Do not resuscitate, Information needs

## Abstract

**Background:**

As the **“**do not resuscitate” (DNR) discussion involves communication, this study explored (1) the effects of a title that included “allow natural death”, and of information contents and outcomes of the decision; and (2) the information needs and consideration of the DNR decision, and benefits and barriers of the DNR discussion.

**Methods:**

Healthy adults (*n* = 524) were presented with a scenario with different titles, information contents, and outcomes, and they rated the probability of a DNR decision. A questionnaire including information needs, consideration of the decision, and benefits and barriers of DNR discussion was also used.

**Results:**

There was a significantly higher probability of signing the DNR order when the title included “allow natural death” (*t* = − 4.51, *p* < 0.001), when comprehensive information was provided (*F* = 60.64, *p* < 0.001), and when there were worse outcomes (*F* = 292.16, *p* < 0.001). Common information needs included remaining life period and the prognosis. Common barriers were the families’ worries and uncertainty about future physical changes.

**Conclusion:**

The title, information contents, and outcomes may influence the DNR decisions. Health-care providers should address the concept of natural death, provide comprehensive information, and help patients and families to overcome the barriers.

## Background

Cardiopulmonary resuscitation (CPR) is an emergency procedure that has been developed to stop cardiopulmonary bypass caused by accidents [1]; however, CPR is not appropriate for patients with a terminal illness or who are dying. It cannot cure the diseases but causes harms [2]. In Taiwan, the Hospice Palliative Act was passed in 2000 and adults can sign a “do not resuscitate” (DNR) order to refuse futile medical treatment when a person is dying.

Some surveys have revealed that most patients are willing to participate in an DNR discussion [3, 4]. However, decision-making is not easy and the timing of the decision may be close to death [5]. While, in Taiwan, terminal patients who received hospice care had to have DNR orders [6], and around 65% patients in intense care units had DNR orders [7]. Less than 2% of the healthy adults had decided DNR orders for themselves, and older adults do not have it [8].

The predictors of a DNR order include older age [9, 10], a cancer diagnosis or poor prognosis [10], poor health condition [9], poor activities of daily living [10], terminal illness with pain symptoms [11], and awareness of prognosis and the survival time following CPR [12]. A study used scenarios with various conditions, and the results showed that patients tended to accept a DNR order under the conditions with older age, pain symptoms, and dementia; and for incurable conditions, better quality of life was an important consideration of DNR [13]. In addition, people with relevant experience, such as experience of caring for patients with a terminal illness and dying patients [14], and those with knowledge [15], tended to sign the DNR order.

The decision of DNR involves specific medical knowledge and legal regulations, and discussing DNR is a process of communication between the patient and physicians. Several variables may influence their understanding. The first variable is the title; using the term “DNR” that includes “do not” may hint that they should give up or that the medical staff should not give any treatment [16]. Meyer (2002) suggested using the term “allow natural death” (AND) to replace DNR, which addresses the fact that death is natural and that the goal of care in the dying phase is for the patient to be comfortable rather than suffering unnecessarily [17]. A study that used scenarios with DNR and AND showed that a higher ratio of nurses and nursing students signed the permissions with AND compared to those with DNR [18]. Physicians and nurses also had more positive perceptions about AND than DNR [19].

The second variable is the contents. The success rate of CPR can influence the participants’ decision; it decreases the decision of receiving CPR when they realize its low success rate [20]. However, people may misunderstand the effect of CPR [21] and do not comprehend the information adequately [22]. In addition, descriptions that are too negative [23] and that have too much or too little information [24] decrease the likelihood of signing the DNR. Few studies have explored the potential effects of the title and contents on the DNR decision in a healthy population.

Better quality of communication about DNR was related to higher satisfaction with care [25]. However, there are problems with the DNR communication: (1) the DNR discussion may be too little and the patients’ preferences may not be respected, (2) the timing of the discussion is too late for the patients to participate, (3) the medical staff do not provide appropriate information for decision-making, and (4) the medical staff inappropriately assume that patients with the DNR orders did not want any treatments [26]. The barriers in patients include that the patients do not understand their diagnosis or prognosis well, are not ready to talk, and are afraid of death [27]. Exploring people’s information needs and their consideration of DNR is important that medical staff could provide the essential information and talk about the individual persons’ needs in relation to DNR decisions.

Therefore, the aims of this study were to explore: (1) how “allow a natural death” versus “do not resuscitate” and specific scenarios and outcomes, influence the willingness to sign a DNR order; and (2) the information needs and consideration of the DNR decisions, and the benefits and barriers of the DNR discussion, in the general population.

## Methods

### Participants and research design

A cross-sectional survey with convenience sampling was conducted. The participants were healthy adults (age > 20 years) who could read and complete the questionnaire. The participants were recruited from the communities and a clinic of family medicine in eastern Taiwan. Ethical approval was obtained before the beginning of the study (Buddhist Tzu Chi General Hospital IRB102–46) and the participants who joined the study gave informed consent before data collection. The participants were given the paper questionnaire and then completed on their own. A research assistant was available to answer questions if necessary.

### Data collection

The data collection included three parts: demographic characteristics and experience of DNR, a scenario, and consideration of the DNR.

#### Demographic characteristics and experience of DNR

The demographic characteristics included gender, age, job, education level, marriage, religion, and self-rated health. Previous experience of DNR was also collected, including if they had heard about DNR, signed a DNR, one day will sign or not, the appropriate timing of discussion, would like to participate in the discussion, and who should make the decision.

#### Scenario

The following scenario was presented: “Mr. W is 80 years old and he was diagnosed with lung cancer five years ago. He has received several surgeries and chemotherapy. There was a period of remission. However, recently, the physician has found that the disease has progressed to stage IV with brain and bone metastasis. His education level is senior high school and his previous work was as a government employee. Since retirement, he has lived with his oldest son who is taking care of him now.” The participants were asked to rate the likelihood of signing the DNR document that had different titles, information contents, and outcomes, by using a scale ranging from 0 (never) to 10 (definitely yes).

##### Title

The official DNR document of the Ministry of Health and Welfare was presented and another document was presented that was exactly the same except for the title being changed to “AND.” Two documents were rated, and in order to balance the sequence effect, a counterbalanced design was used in which half of the participants rated the DNR document first and the other half rated the AND document first.

##### Information contents

The following information was given in sequence: (1) terminal illness: the physician said that the disease has progressed to the end stage and no curative treatment can be provided; (2) goal: the physician said that the goal of DNR is to enable patients to die smoothly and peacefully; (3) success rate: the physician said that the success rate of CPR in patients with a terminal illness is less than 1%, and even though CPR could extend life, there would be only several days of survival time; and (4) harm: the physician said that the CPR procedure includes endotracheal intubation, chest presses, and defibrillation, which may cause harm.

The arrangement of the information was that: bad news was delivered first so that the participants could understand that the disease could not be cured; and then the goal of letting the patient die smoothly and peacefully was presented; in the end, the success rate and side effects of CPR for terminal patients were provided. The participants rated four times when they got information.

##### Outcomes

Different outcomes based on the level of functional impairment and dependence were given. The following consequences of CPR were stated: (1) the patient has to stay in an intensive care unit and he will die within two weeks; (2) the patient has an endotracheal intubation and lives with a ventilator; (3) the patient is in a vegetative state and unconscious; (4) the patient is clearly conscious but cannot take care of himself, and has to rely on others; (5) the patient may have a tracheostomy tube but can take care of himself/herself; and (6) the patient will completely recover.

#### Consideration of the DNR

A standardized survey was used that included the information needs (11 items), the important factors related to the DNR decision (10 items), the benefits of a DNR discussion (five items), the barriers of a DNR discussion with you (seven items), and the barriers of a DNR discussion with families who have a severe disease (eight items). The items were developed from a previous qualitative study [28]. A dichotomous scale (yes/no) was used to rate whether they needed the specific information, and whether the DNR discussion brought the benefits, and whether they had the barriers.

### Statistical analysis

Descriptive statistics were used to present the demographic characteristics and previous experience of DNR. A repeated measures one-way analysis of variance was employed to examine whether there were significant differences in the likelihood of signing the DNR order between the different titles, information contents, and outcomes. The percentage was used to present the importance of the consideration of the DNR.

## Results

A total of 524 participants were recruited. The mean age was 39.19 years (*SD* = 14.34), 312 were female, more than half had a college or university degree, and 39.31% rated their health as good. Regarding DNR experience, 84.16% had heard about DNR, 24.43% had signed a DNR order, 95.23% would like to participate in the DNR discussion, and 50.76% would accept the order one day. Regarding the appropriate timing of the discussion, the first was “when the disease had progressed to the terminal stage” (30.34%) and the second was “when the patient was healthy” (27.48%). In addition, 54.39% agreed that patients and their families should make the DNR decision together and share the responsibility, and 35.50% stated that the patients should make the decision by themselves (see Table 1).

**Table 1:** Demographic characteristics and experience of “do not resuscitate”

Variables	*N* (%)
Age (years)	Mean = 39.19 (*SD* = 14.34, range = 20–88)
Gender	
Male	212 (40.46)
Female	312 (59.54)
Job	
Full-time	327 (62.40)
Part-time	48 (9.16)
None	149 (28.44)
Education	
None	11 (2.10)
Elementary school	29 (5.53)
Junior high school	46 (8.21)
Senior high school	99 (18.89)
College and university	277 (52.86)
Postgraduate	62 (11.83)
Marriage	
Single	222 (42.37)
Married	262 (50.00)
Divorced	19 (3.63)
Widowed	14 (2.67)
Others	7 (1.34)
Self-rated health	
Very good	98 (18.70)
Good	206 (39.31)
Usual	192 (36.64)
Not good	20 (3.82)
Very bad	8 (1.53)
Have heard about DNR?	
Yes	441 (84.16)
No	83 (13.84)
Have had a DNR order?	
Yes	128 (24.43)
No	396 (75.57)
One day, will you accept a DNR order?	
Definitely yes	266 (50.76)
Not sure	241 (45.99)
Definitely no	17 (3.24)
The appropriate timing of discussing DNR	
Healthy	144 (27.48)
Just been diagnosed	68 (12.98)
Disease progressed	105 (20.04)
Terminal stage of the disease	159 (30.34)
Facing death/dying	48 (9.16)
Would you like participate in the DNR discussion?	
Yes	499 (95.23)
No	25 (4.77)
Who should make the DNR decision?	
Self	186 (35.50)
Families	12 (2.29)
Medical staff	5 (0.95)
Self and families	285 (54.39)
Self and medical staff	31 (5.92)
Self, families, and medical staff	5 (0.95)

*DNR* Do Not Resuscitate

Regarding the title, there was a significantly higher probability of signing when using the AND document than when using the DNR document (*t* = − 4.51, *p* < 0.001). Regarding the information contents, there were significant differences in the likelihood of signing the DNR order between the four different information contents (*F* = 60.64, *p* < 0.001), and the post-hoc analysis showed that the higher probability was related to more information. Regarding the outcomes, there were significant differences (*F* = 292.16, *p* < 0.001), and the post-hoc analysis showed that “the patient was in a vegetative state and unconscious” outcome had the highest probability and that “the patient has to stay in an intensive care unit and will die within two weeks” and “the patient has an endotracheal intubation and lives with a ventilator” had the second highest probability. This was followed by “the patient is clearly conscious but cannot take care of himself/herself, and has to rely on others,” “the patient may have a tracheostomy tube but can take care of himself/herself,” and “the patient will completely recover” in sequence (see Table 2).

**Table 2:** The probability of signing a “do not resuscitate” order with a different title, information contents, and outcomes

	Mean (*SD*)	*F*/*p*
Titles		
DNR	7.81 (2.19)	*t* = −4.51,
AND	8.11 (1.96)	*p* < 0.001
Information contents		
(1) terminal illness	7.86 (2.24)	*F* = 60.64,
(1) + (2) goal	8.09 (2.19)	*p* < 0.001
(1) + (2) + (3) success rate	8.52 (2.01)	4 > 3 > 2 > 1
(1) + (2) + (3) + (4) harm	8.62 (2.03)	
Outcomes		
(1) The patient has to stay in an intensive care unit and will die within two weeks.	7.29 (2.51)	*F* = 292.16, *p* < 0.001
(2) The patient has an endotracheal intubation and lives with a ventilator.	7.33 (2.60)	3 > 2 = 1 > 4 > 5 > 6
(3) The patient is in a vegetative state and unconscious.	7.63 (2.82)	
(4) The patient is clearly conscious but cannot take care of himself/herself, and has to rely on others.	6.58 (2.89)	
(5) The patient may have a tracheostomy tube but can take care of himself/herself.	4.96 (2.96)	
(6) The patient will completely recover.	2.76 (3.51)	

*AND* Allowed Natural Death, *DNR* Do Not Resuscitate

The most common information needs were “remaining life period” (75.57%), “the prognosis of the disease” (74.43%), “future symptoms” (70.80%), “hospice and palliative care” (67.56%), “the diagnosis of the disease” (66.60%), and “CPR procedure” (63.93%). The important factors when making the DNR decision included “the consequence of the CPR procedure” (88.93%), “patients’ willingness” (88.55%), “patients’ current physical conditions” (87.40%), “suffering when receiving CPR” (85.88%), and “still having other curative treatments” (83.97%).

The benefits of a DNR discussion included “reducing suffering” (72.90%), “arrangement of medical care as wished” (72.52%), and “reduction of the families’ burden” (70.80%). The barriers related to the participants not wanting to discuss DNR included “families’ worries” (50.95%) and “uncertainty about future physical changes” (46.76%). The barriers related to a discussion about DNR with families who had a severe disease included “worry that they cannot handle the topic” (58.59%), “they do not know when the appropriate timing is” (41.22%), “they do not know how to discuss it with them” (36.64%), and “they do not know the progress of the disease” (35.88%) (see Table 3).

**Table 3:** Information needs, benefits, and barriers of the “do not resuscitate” discussion

	*N* (%)
Information needs	
Remaining life period	396 (75.57)
The prognosis of the disease	390 (74.43)
Future symptoms	371 (70.80)
Hospice and palliative care	354 (67.56)
The diagnosis of the disease	349 (66.60)
CPR procedure	335 (63.93)
Cannot continue curative treatments	292 (55.73)
Dying symptoms and death situation	192 (36.64)
Artificial nutrition	162 (30.92)
New clinical trial	149 (28.44)
Other physicians and hospitals (second opinion)	77 (14.969)
Important factors related to the DNR decision	
The consequence of the CPR procedure	466 (88.93)
Patients’ willingness, such as whether they have expressed an opinion about DNR	464 (88.55)
Patients’ current physical conditions, such as pain, uncomfortable symptoms, and suffering	458 (87.40)
Suffering when receiving CPR	450 (85.88)
Still have other curative treatments, such as operations or medicine	440 (83.97)
The diagnosis of the disease	438 (83.59)
Patients’ age	432 (82.44)
Future care task, such as taking patients to the hospital and care	406 (77.48)
Future care expenditure	387 (73.85)
Other families’ opinions about DNR	281 (53.63)
Benefits of the DNR discussion	
Reduction of suffering	382 (72.90)
Arrangement of medical care as wished	380 (72.52)
Reduction of families’ burden	371 (70.80)
Arrangement of funeral as wished	304 (58.02)
Arrangement of property as wished	214 (40.84)
Barriers that stopped you wanting to discuss DNR	
Families’ worries	267 (50.95)
Uncertainty about future physical changes	245 (46.76)
No one to discuss it with	80 (15.27)
Families’ opinions	64 (12.21)
Worries that physicians gave up treatment	58 (11.07)
Families can understand my ideas without discussion	57 (10.88)
Taboo of death	40 (7.63)
Barriers to the DNR discussion with your family member with advanced disease	
Worry that they cannot handle the topic	307 (58.59)
Do not know when the appropriate timing is	216 (41.22)
Do not know how to discuss it with them	192 (36.64)
Do not know the progress of the disease	188 (35.88)
Should let the medical staff discuss it	153 (29.20)
Worry that they might refuse/reject treatment	136 (25.95)
Worry about the taboo of death	122 (23.28)
Worry about others’ gossip (e.g., property)	96 (18.32)

*CPR* Cardiopulmonary resuscitation, *DNR* Do Not Resuscitate

## Discussion

The study used scenarios to explore the factors related to a DNR decision. It was found that the participants were more willing to accept a DNR decision when the title referred to AND, when there was comprehensive information, and when there were worse outcomes. The participants took into account current physical conditions and future care plans, and they considered the consequences of DNR and the patient’s willingness. More than 70% of participants agreed that DNR could reduce the patient’s suffering and family’s burden. However, family’s worries, uncertainty about the physical conditions, and lack of skills were barriers to the DNR communication.

Similar to a previous study of medical staff [18], the results supported that the general population also prefer the title with AND rather than DNR. The document with “do not” in the title may hint at giving up and the medical jargon may be difficult to understand [14]. In addition, following the promotion of education about life and death and hospice palliative care by the government and hospice-related organizations, the general population can accept that death is natural and face the DNR issue.

The information contents may influence the decision. When the participants had comprehensive information, they had a higher willingness to sign the DNR order. Comprehensive information could include the prognosis of the disease, the goal of DNR, and the success rate and side effects of CPR. The outcome of CPR was one of important considerations [29]. People can evaluate whether the effect of CPR would match their goal and the patient’s best interest. It might decrease the probability of CPR when the participants understand the survival rate of CPR for the dying patient [20]. In addition, they evaluated the level of functional impairment and dependence. If the patient had severe functional impairment and had to depend on others’ care, they then tended to sign the DNR order.

Worse physical conditions and no curative treatment would make people choose DNR, as well as the suffering when receiving CPR. The reason was that if there was no chance to prolong life, then they would not increase the patient’s suffering. If the patients were too old, they also would not want to increase the burden and suffering. In addition, future symptoms and the CPR procedure were regarded as important information about the level of suffering, and they may accept a DNR order if there was too much suffering.

People needed several information for making a DNR decision. The most important information needed for decision-making was “remaining life period,” as has been found for patients with advanced lung cancer [30]. DNR was viewed as a death-related decision, and how much time the patients have was regarded as important information. They also needed information about the disease characteristics of diagnosis and prognosis, which can be used to determine if the patients were in the end-of-life stage. Expectancy about prolonging life was an important foundation of the DNR decision [31].

Patients’ willingness was another important consideration. If the patients had expressed their opinions about DNR and end-of-life medical treatments, people could follow the patients’ willingness. However, if there was no expression, they could make decisions based on only the current information they had, including the physicians’ suggestions, previous understanding of the patient’s values, and the best interest of the patient.

The participants considered that DNR can reduce suffering and the medical care can be arranged as the patients wish. It also decreases the family’s burden, not only in terms of medical care but also the psychological burden, and they did not have to make the decision for the patient.

In spite of the benefits, there were still barriers to the discussion. Discussion of the DNR and end-of-life issues involve various skills. A lack of skill was one of the main barrier to discussion for the families with advanced disease [29]. They worried that they could not handle the topic, and did not know how to open and continue the discussion. In addition, the participants did not want to discuss their own DNR as it could contribute to the families’ worries. The dialogue nature of the DNR discussion is related to death [32], and they were afraid of increasing the families’ worries. On the other hand, they may not understand certain aspects of the physical changes and prognosis, and they did not have enough information about the symptoms and the dying process. Due to the uncertainty, it may be difficult to discuss it and to make a decision. The relevant medical information was also an obstruction that they would like to let the physicians take responsibility for in the discussion.

In the clinical situation, the health-care providers can address the concept of natural death and peaceful dying, and deliver comprehensive information about the goal, likelihood of success of CPR, potential harm, and outcomes. The health-care providers should help people to detect the benefits but also to overcome the barriers in terms of information and discussion skills.

Some limitations of this study should be acknowledged. First, this study used a scenario but there may be a difference between behaviors in real situations and choices in scenarios. Second, the participants had heard about DNR and were willing to participate in the DNR discussion; however, the results may not apply to participants who are not familiar with DNR. Third, the title and information were part of the communication, and other factors, such as the relationships between the families and patients, and between the physicians and patients, were not included in this study. Fourth, convenience sampling was used that the participants who were willing to join the study may have positive attitude about DNR and hospice care, and the results may not be generalizable beyond Taiwan. In future studies, an experimental design with a different title and different information could be conducted in clinical practice to exam whether the patients receiving AND tend to accept the order or have higher satisfaction.

## Conclusion

Discussion about DNR is a process of communication, and the title that includes AND, comprehensive information about terminal illness, the goal of DNR, and the success rate and harm of CPR, and the worse outcomes of CPR with severe functional impairment and dependency may increase the DNR decision. The healthy adults needed information about the remaining life period, the prognosis of the disease, and future symptoms, the consequence of the CPR procedure and suffering when receiving CPR, and the patients’ willingness. The barriers arising from the families’ worries, uncertainty about future physical changes, and lack of skills may disrupt the DNR discussion.

## Abbreviations

AND: Allow natural death
CPR: Cardiopulmonary resuscitation
DNR: Do not resuscitate
